# Adult coronary artery bypass grafting by congenital surgeons—a propensity matched analysis

**DOI:** 10.1093/ejcts/ezab081

**Published:** 2021-02-15

**Authors:** Filippo Rapetto, Vito D Bruno, Cha Rajakaruna, Alan J Bryan, Andrew J Parry, Massimo Caputo, Serban C Stoica

**Affiliations:** 1 Department of Cardiac Surgery, Bristol Heart Institute, Bristol, UK; 2 Department of Cardiac Surgery, Bristol Royal Hospital for Children, Bristol, UK

**Keywords:** ACHD, CABG, Volume–outcome effect

## Abstract

**OBJECTIVES:**

Surgical myocardial revascularization will be increasingly needed in adult patients with congenital heart disease. We investigated the results of coronary artery bypass grafting (CABG) performed on adults by congenital cardiac surgeons at our institution.

**METHODS:**

We conducted a retrospective, single-centre study. Adults undergoing isolated or combined CABG from 2004 to 2017 were included. Early and late outcomes were analyzed for the whole cohort. Furthermore, a propensity matched analysis was conducted comparing the results of isolated CABG between congenital and adult surgeons.

**RESULTS:**

A total of 514 and 113 patients had isolated and combined CABG for acquired heart disease, respectively. A total of 33 patients had myocardial revascularization at the time of surgery for congenital heart disease. Overall early mortality was 1.2%, the rate of re-exploration for bleeding was 4.5%, and an internal mammary artery to left anterior descending artery graft was used in 85.6% patients. One-year survival was 97.5% (96.2–98.8%), and 5-year survival was 88.0% (84.8–91.3%). After propensity matching (468 pairs), early mortality (0.6% vs 1.2%, *P* = 0.51), re-exploration for bleeding (3.6% vs 3.0%, *P* = 0.72), use of internal mammary artery to left anterior descending artery graft (92.7% vs 91.9%, *P* = 0.70) and late survival did not differ between congenital surgeons and adult surgeons, respectively.

**CONCLUSIONS:**

Surgical myocardial revascularization can be required for adult congenital patients in a broad spectrum of clinical situations. Despite lower volumes, congenital cardiac surgeons perform CABG safely and with results that are comparable to those of the adult surgeons at our centre.

**Figure:**
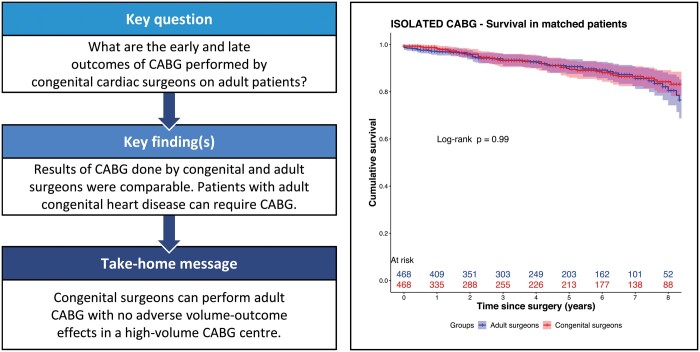

## INTRODUCTION

The management of children with congenital heart disease (CHD) has improved dramatically over the last few decades; consequently, an increasing number of congenital patients will require surgical procedures as adults [1, 2]. As a result of the improved life expectancy, the incidence of concomitant coronary artery disease (CAD) in this population is rising [3, 4], and the need for myocardial revascularization at the time of surgery is an additional challenge. Furthermore, a number of congenital conditions such as coronary anomalies may be treated with surgical revascularization in selected cases [5].

Coronary artery bypass grafting (CABG) is the most common heart operation performed worldwide [6]; nevertheless, it is not undertaken routinely by congenital cardiac surgeons in many centres.

Our aims were to retrospectively analyze the results of CABG performed on adult patients by congenital cardiac surgeons at our institution, using widely recognized clinical outcomes as quality indicators [6, 7]. Furthermore, we conducted a propensity matched analysis to compare the results of isolated CABG between congenital surgeons and high-volume coronary surgeons at the same centre.

## PATIENTS AND METHODS

This is a retrospective, single-centre study. The study was conducted in accordance with the principles of the Declaration of Helsinki; it was approved by the local institutional board and the requirement for individual patient consent was waived.

Standard data are collected prospectively for all patients undergoing cardiac surgery at the Bristol Heart Institute. The data collection form includes 5 sections that are filled in consecutively by an anaesthetist, a surgeon, a perfusionist and nurses; data are entered into a database (Patient Analysis & Tracking System, Dendrite Clinical Systems, Henley-on-Thames, UK).

Inclusion criteria for this study were age >18 years and CABG surgery (isolated or combined); patients with acquired or CHD were included as long as the responsible surgeon was a congenital cardiac surgeon. All congenital surgeons who performed operations included in this study had been fully trained in adult cardiac surgery and had worked as adult cardiac surgeons before developing a special interest in congenital surgery. Data for 660 consecutive patients between July 2004 and December 2017 were obtained from the database.

The logistic EuroSCORE [8] was calculated for all patients. Early (30-day) mortality, reoperation for bleeding, use of an internal mammary artery (IMA) graft to the left anterior descending (LAD) artery, use of at least 1 arterial graft and long-term survival were used as quality indicators.

As a first step, we analyzed the early and late outcomes considering all patients, irrespective of the baseline diagnosis.

To assess the congenital surgeons’ CABG outcomes more specifically and to reduce the potential negative effects of confounders caused by a heterogeneous study population, 514 patients who underwent isolated CABG were selected from the whole cohort. This subgroup was then propensity matched with a group of 9618 consecutive patients who had isolated CABG done by adult cardiac surgeons over the same period and at the same institution.

### Statistical analysis

Continuous variables are reported as mean ± 1 standard deviation. Categorical variables are reported as percentage frequencies. Actuarial long-term survivals are presented as Kaplan–Meier curves, and the log-rank test was used to compare the curves.

The annual volume for isolated CABG was calculated for each surgeon (adult and congenital) included in the study: the total number of cases done by a surgeon was divided by the number of months that surgeon worked at our institution during the study period, thus obtaining the average isolated CABGs per month. This was then multiplied by 12 in order to calculate the average annual volume.

A propensity score matched analysis was conducted to compare results in isolated CABG between congenital and adult cardiac surgeons. The main preoperative characteristics (age, gender, hypertension, left ventricular function, recent myocardial infarction, pulmonary disease, extracardiac arteriopathy, atrial fibrillation, NYHA class, Canadian Cardiovascular Society class, smoking habit, insulin-dependent diabetes mellitus, urgent/emergent surgery, logistic EuroSCORE) were included in the matching. The nearest neighbour method was used, and the balance after matching was evaluated with standardized mean differences. After 1:1 propensity score matching, variables were compared using paired Wilcoxon test for continuous variables and McNemar test for dichotomous variables. All tests were two-sided with a level set at 0.05 for statistical significance. No correction for multiple testing was undertaken. Clinical data were recorded and subsequently tabulated with Microsoft Excel (VR Microsoft Corp, Redmond, WA, USA). The statistical analysis was computed using RStudio version 1.2.5042 (RStudio: Integrated Development for R. RStudio, Inc., Boston, MA, USA). The propensity score matching was computed with the MatchIt package.

## RESULTS

### Unmatched analysis

**Figure 1:**
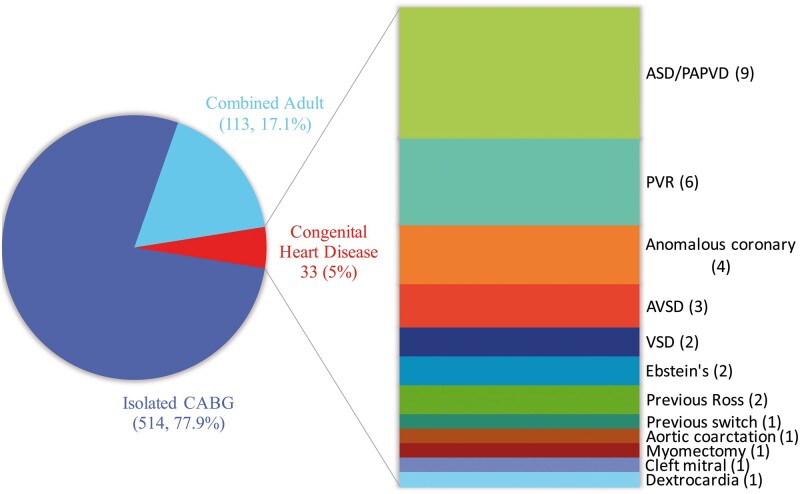
Case mix for patients undergoing isolated or combined coronary artery bypass grafting performed by congenital surgeons over the study period. Congenital diagnosis/procedure is shown in detail for patients with a history of congenital heart disease (number of patients in brackets). ASD: atrial septal defect; AVSD: atrioventricular septal defect; CABG: coronary artery bypass grafting; PAPVD: partial anomalous pulmonary venous drainage; PVR: pulmonary valve replacement.

The baseline patient characteristics for all patients and for patients with concomitant CHD are summarized in Table 1. Over the study period, 660 patients underwent CABG performed by congenital cardiac surgeons. A total of 514 (77.9%) patients underwent isolated CABG and 113 (17.1%) patients underwent combined CABG in the setting of acquired heart disease; the remaining 33 (5%) patients had concomitant CABG at the time of surgery for CHD. Within this last group, the most frequent procedures were atrial septal defect/partial anomalous pulmonary venous drainage repair in 9 (27.3%) patients, pulmonary valve replacement in 6 (18.2%) and surgery for coronary artery anomalies in 4 (12.1%). The case mix is illustrated in Fig. 1.

**Table 1:** Baseline patient characteristics for coronary artery bypass grafting done by congenital surgeons

	All patients (*n* = 660)	ACHD patients (*n* = 33)
Age (years), mean ± SD	66.6 ± 10.3	56.2 ± 16.3
Male gender, *n* (%)	545 (82.6)	21 (63.6)
Hypertension, *n* (%)	456 (69.1)	11 (33.3)
IDDM, *n* (%)	46 (7.0)	1 (3.0)
Current smoker, *n* (%)	77 (11.7)	3 (9.1)
Pulmonary disease,^a^*n* (%)	90 (13.6)	4 (12.1)
Extracardiac arteriopathy,^a^*n* (%)	66 (10.0)	1 (3.0)
Atrial fibrillation, *n* (%)	49 (7.4)	7 (21.2)
NYHA class III–IV, *n* (%)	178 (27.0)	14 (42.4)
Angina CCS class III-IV, *n* (%)	254 (38.5)	3 (9.1)
Neurological dysfunction,^a^*n* (%)	9 (1.4)	0
Recent MI (<90 days), *n* (%)	237 (35.9)	4 (12.1)
LVEF <50%, *n* (%)	183 (27.7)	15 (45.4)
LVEF <30%, *n* (%)	38 (5.7)	2 (6.0)
Previous sternotomy, *n* (%)	16 (2.4)	8 (24.2)
Non-elective surgery, *n* (%)	409 (62.0)	5 (15.1)
Logistic EuroSCORE, mean ± SD	5.2 ± 6.6	6.7 ± 8.9

ACHD: adult congenital heart disease; CCS: Canadian Cardiovascular Society; IDDM: insulin-dependent diabetes mellitus; LVEF: left ventricular ejection fraction; MI: myocardial infarction; NYHA: New York Heart Association; SD: standard deviation.

a As defined by logistic EuroSCORE [8].

**Figure 2:**
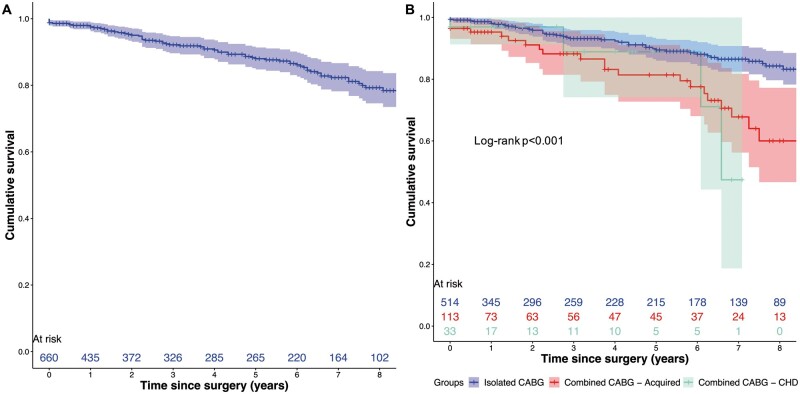
Actuarial survival curves (**A**) for the whole study cohort and (**B**) stratified for procedure (isolated coronary artery bypass grafting versus combined coronary artery bypass grafting in acquired disease versus combined coronary artery bypass grafting in congenital heart disease). CABG: Coronary artery bypass grafting; CHD: congenital heart disease.

Considering the whole cohort, 8 (1.2%) patients died before discharge from hospital; the observed/expected mortality calculated on the logistic EuroSCORE was 0.23. The rate of re-exploration for bleeding was 4.5%. An IMA graft to the LAD artery was used in 565 (85.6%) patients and an arterial graft was used in 601 (91.1%) patients. One-year survival was 97.5% [95% confidence interval (CI) 96.2–98.8%] and 5-year survival was 88.0% (95% CI 84.8–91.3%). Considering the 33 patients with CHD, hospital mortality was 3.0% (1/33 patients) and no patient was re-explored for bleeding. An IMA to LAD graft and an arterial graft were used in 12 (36.3%) and 13 (39.4%) patients, respectively. One-year survival was 97.0% (95% CI 91.3–100%) and 5-year survival was 88.9% (95% CI 74.2–100%). Figure 2A and B illustrates the actuarial survival for the whole group and stratified according to isolated CABG, combined CABG in patients with acquired disease and combined CABG in patients with CHD.

### Propensity score matching

**Figure 3:**
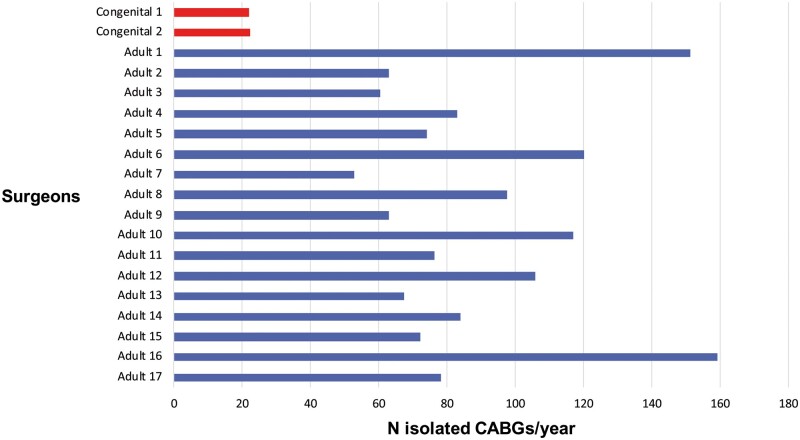
Annual volume for isolated coronary artery bypass grafting for all surgeons included in the analysis. Not all adult surgeons worked at our institution for the whole study period. CABG: coronary artery bypass grafting.

Figure 3 illustrates the isolated CABG annual volume for congenital and adult surgeons included in the analysis throughout the study period. After propensity matching, each group was composed of 468 patients who underwent isolated CABG. The surgical risk as estimated by logistic EuroSCORE was overall relatively low (<5 in both groups); baseline characteristics and outcomes of the matched patients are summarized in Table 2. There were no statistically significant differences between congenital surgeons’ and adult surgeons’ results in terms of early mortality, re-exploration for bleeding and use of an IMA to LAD graft (0.6% vs 1.2%, *P* = 0.51; 3.6% vs 3.0%, *P* = 0.72; 92.7% vs 91.9%, *P* = 0.70 respectively). The use of any arterial graft was higher in patients operated on by congenital surgeons (98.3% vs 95.7%, *P* = 0.031). Regarding late survival, no statistically significant differences were found. One-year survival was 98.2% (95% CI 96.9–99.6%) in patients operated on by congenital surgeons and 97.0% (95% CI 95.4–98.5%) in patients operated on by adult surgeons; 5-year survival was 89.5% (95% CI 86.0–93%) and 90.6% (95% CI 87.6–93.8%), respectively.

**Table 2:** Baseline patient characteristics and clinical outcomes for isolated coronary artery bypass grafting after 1:1 propensity matching

	Congenital surgeons (*n* = 468)	Adult surgeons (*n* = 468)	*P*-value	SMD
Baseline patient characteristics				
Age (years), mean ± SD	66.6 ± 9.3	66.5 ± 9.2	0.78	0.01
Male gender, *n* (%)	402 (85.9)	403 (86.1)	1	0.01
Hypertension, *n* (%)	345(73.7)	343 (73.3)	0.94	0.01
IDDM, *n* (%)	34 (7.3)	36 (7.7)	0.90	<0.01
Current smoker, *n* (%)	63 (13.5)	62 (13.2)	1	<0.01
Pulmonary disease,^a^*n* (%)	67 (14.3)	58 (12.4)	0.44	0.03
Extracardiac arteriopathy,^a^*n* (%)	44 (9.4)	42 (8.9)	0.91	<0.01
Atrial fibrillation, *n* (%)	13 (2.8)	7 (1.5)	0.26	<0.01
NYHA class III–IV, *n* (%)	104 (22.2)	93 (19.9)	0.44	0.02
Angina CCS class III–IV, *n* (%)	205 (43.8)	202 (43.2)	0.90	0.02
Recent MI (<90 days), *n* (%)	225 (48.1)	229 (48.9)	0.84	0.02
LVEF <50%, *n* (%)	114 (24.4)	116 (24.7)	0.94	0.02
Non-elective surgery, *n* (%)	317 (67.7)	315 (67.3)	0.94	<0.01
Logistic EuroSCORE, mean ± SD	4.3 ± 5.2	4.5 ± 5.7	0.99	0.1
Outcomes				
30-Day mortality, *n* (%)	3 (0.6)	6 (1.2)	0.51	
Re-exploration (bleeding), *n* (%)	17 (3.6)	14 (3.0)	0.72	
IMA to LAD graft, *n* (%)	434 (92.7)	430 (91.9)	0.70	
Any arterial graft, *n* (%)	460 (98.3)	448 (95.7)	0.031*	
1-Year survival (95% CI)	98.2% (96.9–99.6%)	97.0% (95.4–98.5%)		
5-Year survival (95% CI)	89.5% (86.0–93.1%)	90.6% (87.6–93.8%)		

CI: confidence interval; CCS: Canadian Cardiovascular Society; CHD: congenital heart disease; IDDM: insulin-dependent diabetes mellitus; IMA: internal mammary artery; LAD: left anterior descending; LVEF: left ventricular ejection fraction; MI: myocardial infarction; NYHA: New York Heart Association; SD: standard deviation; SMD: standardized mean difference.

a As defined by logistic EuroSCORE [8].

* Statistically significant.

## DISCUSSION

In this retrospective study, we analyzed the results of CABG surgery performed by congenital cardiac surgeons at a single institution. Three main factors represent the background for our investigation. First, as a consequence of the improved survival of patients with CHD, the incidence of CAD in this specific population is increasing [3, 4] and a parallel increase in the need for CABG can be postulated. Second, in most centres, congenital cardiac surgeons do not routinely undertake CABG, and their outcomes in coronary surgery are not known. Third, congenital cardiac surgeons at our centre have maintained a low-volume CABG practice even on patients without any history of CHD; the care for these patients is delivered in the context of a high-volume adult cardiac surgical unit with extensive expertise in CABG (about 600 isolated CABGs/year over the study period).

Our study has 2 main findings. First, CAD requiring surgical revascularization can develop in a broad spectrum of adult congenital heart disease (ACHD). Over the 14-year study period, 33 patients with 12 different congenital diagnoses needed surgical myocardial revascularization at the time of surgery for their baseline pathology. The intersection between ACHD and risk factors for acquired cardiovascular disease has now been acknowledged in the scientific literature [3, 9, 10]; nevertheless, the prevalence of significant CAD in specific pathologies is not fully known [11]. Few surgical series have been published to date investigating CABG in the context of ACHD, with baseline characteristics and outcome comparable to ours [12, 13].

Second, congenital cardiac surgeons can perform CABG safely and with results that are comparable with those of the adult cardiac surgeons at our centre. To investigate congenital surgeons’ performance in coronary surgery, we selected patients who underwent isolated CABG and we matched them with patients operated on by adult surgeons over the same period and at the same institution. Of note, patients were referred from the same catchment area, and about 2/3 operations in this series were performed urgently or emergently from a pool of patients who had been referred to the centre, not to a specific surgeon. Patients were treated by the same anaesthetists, intensivists and nurses in the same Intensive Care Unit and ward, regardless of the surgical team who performed the procedure.

The volume–outcome relationship in cardiac surgery has been extensively investigated [14–19]; intuitively, high-volume surgeons are expected to have better results than low-volume surgeons for a given procedure. However, the demonstration of this concept has not been straightforward in the field of coronary surgery. The European Society of Cardiology (ESC)/European Association for Cardio-Thoracic Surgery (EACTS) guidelines recommend that CABG should ‘be performed at institutions with annual institutional volumes of ≥200 CABG cases’; however, a cut-off for individual surgeons has not been set [20]; the document also suggests that being able to rescue patients from complications (a typical feature of high-volume centres) and meeting pre-set quality indicators may be more important than individual surgeons’ volume [17, 21]. The American Heart Association (AHA)/American College of Cardiology (ACC) guidelines include similar recommendations, stressing the importance of participating ‘in a state, regional, or national clinical data registry and […] receive periodic reports of […] risk-adjusted outcomes’ [22].

The similar early and late results observed after propensity matching in our 2 groups could be a further demonstration that outcomes in coronary surgery are more system related than surgeon related. Possibly, the combination of a number of other factors such as surgical indication, timing of surgery, antiplatelet therapy management, blood product management, intensive care and postoperative early mobilization outweighs the impact of the single surgeon on the final outcome.

The findings of our study can also be the background for further discussions. Even though concomitant CAD and ACHD will become more and more common, in our series, only 33 adult patients with CHD over a period of 14 years needed surgical revascularization. In some of these cases, CABG had to be performed in a redo setting and/or as a bailout; notably, almost 25% of patients with ACHD who needed CABG in this study had undergone previous sternotomy. Therefore, a small but regular CABG practice is entirely optional for congenital surgeons and probably rare. In our opinion, where it can be accommodated, this practice can be useful to maintain microvascular skills that will be increasingly needed. Furthermore, in our experience, it has proven beneficial in a smaller number of cases where CABG was required in paediatric patients, a clinical scenario that will likely become relatively common [23]; Table 3 illustrates 5 paediatric cases from the study period that had to be treated with CABG (not included in the analysis as age <18).

**Table 3:** Paediatric patients requiring coronary artery bypass grafting over the study period (excluded from the analysis)

	Age	Diagnosis	Operation
Patient 1	13 years	Anomalous left coronary artery running between aorta and main pulmonary artery. Out of hospital cardiac arrest	Off-pump CABG × 1 (LIMA–LAD)
Patient 2	6 years	Critical aortic stenosis. Cardiac arrest and RCA ischaemia after Ross procedure	Ross procedure CABG × 1 (SVG–RCA)
Patient 3	11 years	Neonatal aortic endocarditis Previous Ross–Konno operation RCA ischaemia (stable angina)	CABG × 1 (LIMA–RCA)
Patient 4	15 years	Left main stem proximal long stenosis and distal aneurysm. Out of hospital cardiac arrest	CABG × 2 (LIMA–LAD, SVG–OM)
Patient 5	Neonate	Taussig–Bing anomaly. ECMO and RCA ischaemia after arterial switch operation	CABG × 1 (RIMA–RCA)

CABG: coronary artery bypass grafting; ECMO: extracorporeal membrane oxygenation; LAD: left anterior descending; LIMA: left internal mammary artery; OM: obtuse marginal; RCA: right coronary artery; RIMA: right internal mammary artery; SVG: saphenous vein graft.

Our data are not sufficient to state that our work model can be successfully exported to any other unit and making such a statement was not within our aims. Clearly, different models may be adopted with good results; for example many congenital departments work in close collaboration with adult units and experienced coronary surgeons are often easily available if required. The scope of our work was mainly to validate our set-up and to acknowledge that, in a minor though significant number of cases, coronary surgery has to be performed in the context of CHD. We do believe this latter aspect has been under-investigated and that our results can trigger an interesting discussion within the cardiac surgical community.

### Limitations

Our study is limited by its retrospective, single-centre design. Although we performed propensity matching to compare our groups, this was not a randomized study and there might be residual unmeasured confounders. Based on the wider context and these initial findings, a randomized study is however not justified or practical. Moreover, the isolated CABG patients that were matched in our analysis represent a relatively low-risk but overall typical cohort. It is therefore possible that our conclusions cannot be extended to a higher-risk CABG population. We defined risk using the mean EuroSCORE and the matched cohorts included high-risk patients. Higher risk usually comes from comorbidities, and it would be fair to presume that, within the same system of care, the results in higher risk cohorts would remain comparable; however, this is not a concept that we intended to test. Finally, although we chose several strong quality indicators for our analysis, data about other factors such as postoperative sternal wound infection, stroke and acute kidney injury were not fully available and have not been included.

## CONCLUSION

Surgical myocardial revascularization can be required for adult congenital patients in a number of different situations. Despite the lower volume, congenital cardiac surgeons perform CABG safely and with results that are comparable to those of the adult surgeons at our institution.

## Funding

No funding was received to conduct this study.

**Conflict of interest:** none declared.

## Author contributions

**Filippo Rapetto:** Conceptualization; Formal analysis; Methodology; Writing—original draft; Writing—review & editing. **Vito D. Bruno:** Formal analysis; Methodology; Validation. **Cha Rajakaruna:** Validation. **Alan J. Bryan:** Conceptualization; Methodology; Supervision; Validation. **Andrew J. Parry:** Supervision; Validation. **Massimo Caputo:** Supervision; Validation. **Serban C. Stoica:** Conceptualization; Methodology; Supervision; Validation; Writing—original draft.

## Reviewer information

European Journal of Cardio-Thoracic Surgery thanks Hani Najm, Alister Joseph Thomas and the other, anonymous reviewer(s) for their contribution to the peer review process of this article.

## Notes

Presented at the 34th Annual Meeting of the European Association for Cardio-Thoracic Surgery, Barcelona, Spain, 8–10 October 2020.

## ABBREVIATIONS

ACHD: Adult congenital heart disease
CABG: Coronary artery bypass grafting
CAD: Coronary artery disease
CHD: Congenital heart disease
CI: Confidence interval
IMA: Internal mammary artery
LAD: Left anterior descending
